# Substantiation of 25 kGy radiation sterilization dose for banked air dried amniotic membrane and evaluation of personnel skill in influencing finished product bioburden

**DOI:** 10.1007/s10561-014-9433-1

**Published:** 2014-03-28

**Authors:** Nagi Marsit, Samira Dwejen, Ibrahim Saad, Sedigh Abdalla, Arej Shaab, Salma Salem, Enas Khanfas, Anas Hasan, Mohamed Mansur, Mohamed Abdul Sammad

**Affiliations:** Tissue Banking Research Group, Biotechnology Research Center, Tweisha, Libyan Authority for Research, Science and Technology, P. O. Box 30313, Tripoli, Libya

**Keywords:** Amniotic membrane, Bioburden, Verification dose, Radiation sterilization dose, Substantiation of 25 kGy

## Abstract

Preparation of amniotic membrane (AM) by air drying method followed by radiation sterilization is simple and valuable approach; sterility and quality of the final AM product are depending on the quality management system at the tissue bank. Validation and substantiation of radiation sterilization dose (RSD) for tissue allografts is an essential step for the development and validation of the standard operating procedures (SOP). Application of SOP is perfectly relying on trained staff. Skills differences among personnel involved in AM preparation could have an effect on microbiological quality of the finished product and subsequently on the RSD required. AM were processed by four different couples of the tissue bank technicians. The AM grafts were randomly selected and subjected to bioburden test to validate and substantiate the 25 kGy RSD. Bioburden test for AM grafts were also useful to evaluate the skill of the tissue bank technicians and thus, to validate the current SOP for air dried AM. Moreover, the effect of placental source on bioburden counts on AM grafts was assessed. Substantiation of the 25 kGy RSD at a sterility assurance level of 10^−1^, and sample item portion = 1, was carried out using Method VD_max_^25^ of the International Organization for Standardization, document no. 11137-2 (ISO in Sterilization of healthcare products—radiation—part 2: establishing the sterilization dose, Method VDmax—substantiation of 25 kGy or 15 kGy as the sterilization dose, International Standard Organization, 2006). The results showed that there were no significant differences in the bioburdens of the four batches (α = 1 %), this means no significant differences in the skill of the four couples of the tissue bank technicians in terms of their ability to process AM according to the air dried AM SOP. The 25 kGy RSD was validated and substantiated as a valid sterilization dose for the AM prepared with the current established SOP at the Biotechnology Research Center experimental tissue bank. The donor’s type of delivery, normal or caesarean, showed no significant effect on the levels of microbial counts on the tested AMs (α = 1 %).

## Introduction

Tissue banking activity and radiation sterilization of tissue allografts has been expanding to many developing countries in the new millennium. The typical operation of a tissue bank to produce sterile tissue allografts necessitates the adoption of any of the tissue banking standards, i.e. American Association of Tissue Banks AATB (AATB 2002), European Association of Tissue Banks (EATB), (EATB 1995), International Atomic Energy Agency (IAEA) standards (IAEA 2003), as well as International Standard Organization ISO documents for radiation sterilization dose (RSD) selection (ISO 11137-1 2006).The confirmation of sterility, safety and efficiency of tissue allografts together with the dose setting and choice of the RSD are the responsibility of the tissue banker (Morales Pedraza et al. 2012).

Human amniotic membranes (AM) is obtained from placentae of healthy mothers upon delivery; it is readily available, cost effective and contains a variety of growth factors that are required for fast healing of burns, ulcers of skin and eye surface. The recent wide use of AM especially in burn and ophthalmology practice imposes an obligation on tissue banks to ensure quality of their product materials.

Our staff gained experience in the past years through the IAEA technical assistance by participating in the IAEA regional and interregional tissue banking activities, namely fellowship programmes, training courses, workshops etc. A project for processing of air dried AM was then proposed in early 1999 in Tajoura Nuclear Research Center (TNRC). Later on, in 2002, a research tissue bank is started at the Human Tissues Department at the Biotechnology Research Center (BTRC). Early batches of AM grafts were utilized in selected burn cases in 2003 (Marsit et al. 2006). Interruptions in the procurement and processing had delayed the extensive use of amnion untill 2009 when the project was revived. During the bank set up phase, AM was prepared by air drying method followed by radiation sterilization. This simple and valuable approach was easily adopted by the experimental tissue banks especially in developing countries; while sterility and quality of the final AM graft were ensured through the quality system established by the tissue bank. However, at international level, distinct developments have been taking place in recent years to standardize the processing procedures and facilitate the selection of the terminal RSD for tissue allografts (Morales Pedraza et al. 2012).The use of 25 kGy as a terminal sterilization dose for tissue allografts has been practiced for several decades since its first introduced in 1950s. The 25 kGy dose is also recommended by the IAEA Standards for Tissue Banks (IAEA 2003) and the AATB Standards (AATB 2002) as the minimum dose for bacterial sterilization, till date it is still acceptable and usable in many tissue banks (Nguyen et al. 2007).

To ensure safety and quality of tissue grafts to be used in clinical applications, retraining is mandatory for personnel involved in any parts of the tissue banking professional activity, especially when there is a need for change in procedures or new scientific knowledge (Kaminski et al. 2013). At the BTRC experimental tissue bank, the standard operating procedures SOP established for processing air dried AM was amended in line with the improvement of the tissue bank capabilities over time, likewise, tissue bank processing staff were retrained in the aseptic technique and processing skills. In addition, the skill of the tissue bank staff is among the factors that might affect the bioburden of the prepared amniotic membrane grafts, thus, control of contamination sources, strictly follow processing procedures and validation of the product samples are crucial and subject to continuous improvement.

The tissue banking standards published by authorized organizations and associations such as ISO, AATB, obliging their member tissue banks to standardize and validate the processing procedures of the produced tissues, and every individual tissue bank is obliged to create their own SOP starting from donor screening and consent till the final step of tissue distribution. In addition, tissue banking experts urge on performing RSD validation using the IAEA code of practice (IAEA 2007) or any of the appropriate ISO documents at least four times a year in order to ensure sterility of the processed tissue allografts, thereof, substantiation of the RSD for air dried AM is essential. The ISO 11137-2 VD_max_^25^ method is considered suitable for AM grafts, as this method is formulated for small batches of biological products (Yusof 2007).

The aim of this work was to validate and substantiate the 25 kGy RSD for air dried AM prepared at the BTRC tissue bank, and to evaluate the differences in microbiological quality of the AM grafts produced by different tissue bank workers. The effect of the types of delivery, either normal or caesarean, on the bioburden level was also investigated.

## Materials and methods

### Human AM preparation procedures

Human amniotic membrane was prepared according to the procedure described in the IAEA Multimedia Distance Learning Package on Tissue Banking, (IAEA 1997) with modification. The SOP was validated by the BTRC experimental tissue bank. More than 235 AM grafts were produced from 91 placentae in the period between January 2009 and November 2010. The AM processing were carried out by four different couples of tissue bank workers; the participating technicians have similar academic background and training levels.

Briefly, human placentas were collected from Maternity Department of Ali Omar Asskar Hospital. The mother donors were screened negative for HIV, HBV and HCV. The adopted processing procedures for air dried amnion were comprised of wet and dry processing stages (IAEA 1997). It is advised to separate processes involving the use of washing solutions and raw tissue allografts from those having adequately clean handling such as cutting, packaging and labeling, for the purpose of minimizing contamination. Each placenta was processed separately, pooling of placentae or AMs together is forbidden.

### Wet processing

At the tissue bank, the AM was peeled off from the placenta; the membranes were thoroughly washed in cold running water till they were free of blood clots. Then, the membranes were washed subsequently three times in sterile saline solution followed by a single wash in 0.05 % sodium hypochlorite solution and finally rinsed three times with sterile distilled water. Washing was done using electrical shaker (GFL 3006, Germany) in screw capped media bottles (DURAN 10011391, Duran Gm bH, Germany) containing 300 ml each; every individual washing/shaking lasted for 15 min. Sterile long forceps were used to transfer AM to washing bottles.

### Dry processing

**Fig. 1:**
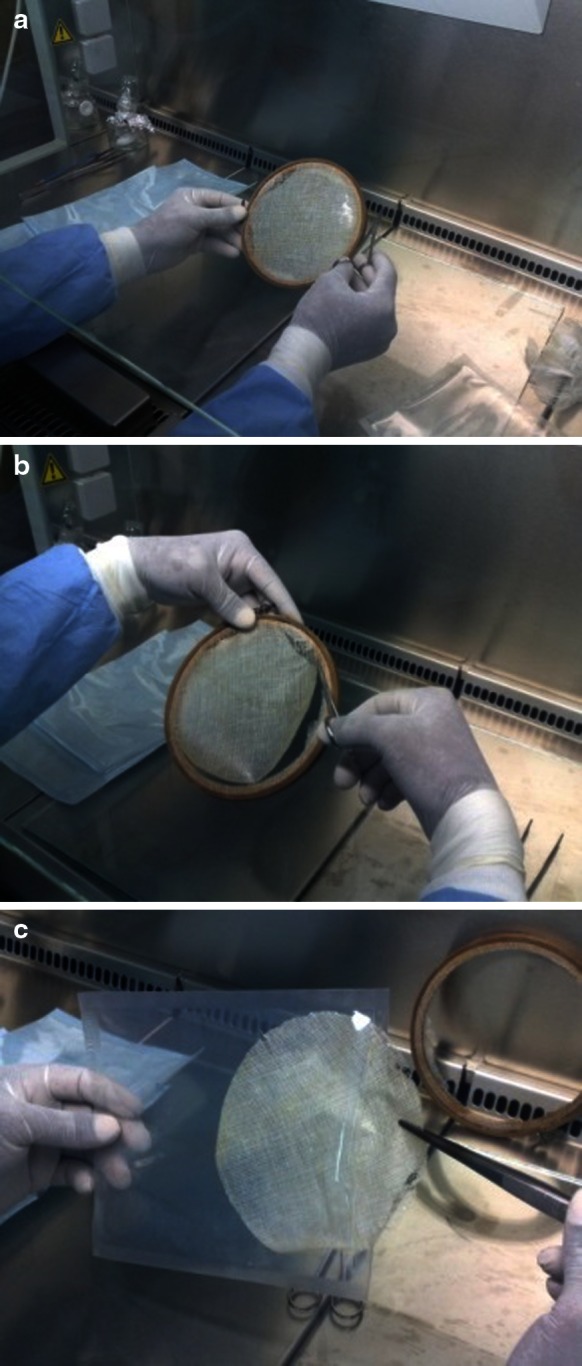
**a** Amniotic membrane stretched over sterile gauze and tightened into sterilized dual circular wooden frame. **b**, **c** Amniotic membrane graft cutting, trimming and packaging in inner polyethylene packs

Subsequently, inside the Class II safety cabinet (HERASAFE KS9, Thermo Scientific, Germany), the membranes were spread over sterile cotton gauze (Winner industries, Shenzhen Co., LTD, China) on a sterile glass plate and sterile saline was used to prevent amnion sticking to the glass plate. The gauze with amnion was then mounted onto sterilized dual circular wooden frames (Ø = 16 cm), “*the ones used in textile stitching crafts*” (Fig. 1a) and air dried in biosafety cabinet for overnight (≥18 h), then, the membranes were cut, trimmed and packed in inner polyethylene packs (Fig. 1 **b**, **c**) which were enveloped in pre-autoclaved self-seal sterilization pouches (Sigma medical supplies corp., Taiwan). The AM grafts envelopes were then labeled, heat sealed using (ALLPAX PT-MJ-4 DS, Gm bH&Co. KG, Germany) machine and stored at room temperature, small pieces remained after cutting was used as quality control samples.

### Validation and substantiation of 25 kGy RSD

#### Obtaining AM samples

The selection of AM graft samples for bioburden test and verification of sterilization dose was done randomly from the stocks of the AM grafts routinely processed during 2009–2010. The collection of 10 AM grafts for bioburden estimation and 10 for verification of dose would not be possible from a single placenta, therefore only months (batches) that were having more than 20 AMs were included in this work.

Although, this approach is not completely comply with the standards for obtaining the samples for bioburden and verification experiment, this modification was unavoidable to overcome constraints set by the standards and was vastly discussed by other authors in previous works (Hilmy et al. 2000; Nguyen et al. 2007). The number of placentae used to collect 20 AM product units for every batch is shown in Table 1.

**Table 1:** Placentae sources, bioburden counts and average grafts produced per placenta of the four batches of amniotic membrane grafts used in bioburden determination for VD_max_^25^ Method

Batch code	*Placenta No.	Type of delivery	Av. grafts/placenta	Bioburden on AM graft (cfu)	Av. Bioburden (cfu) ± SD
A Jan 2009 (4 Placentae)	5	N	4.2	9	3.9 ± 2.8
	4	N		6	
	3	CS		5	
	1	CS		6	
	1	CS		1	
	1	CS		2	
	1	CS		4	
	1	CS		0	
	4	N		5	
	4	N		1	
B Feb 2009 (4 Placentae)	19	CS	3.5	10	13.9 ± 5.9
	19	CS		14	
	18	CS		14	
	16	N		26	
	15	N		10	
	15	N		12	
	16	N		11	
	15	N		21	
	16	N		5	
	18	CS		16	
C Jan 2010 (6 Placentae)	41	CS	3.0	10	46.8 ± 58.1
	41	CS		13	
	39	CS		21	
	39	CS		17	
	38	CS		11	
	37	N		7	
	36	CS		114	
	36	CS		160	
	36	CS		112	
	35	CS		3	
D Oct 2010 (7 Placentae)	87	CS	3.6	14	7.8 ± 7.6
	86	N		6	
	88	CS		0	
	83	N		5	
	85	N		19	
	84	N		22	
	84	N		3	
	86	N		3	
	91	N		3	
	84	N		3	
Overall average cfu					18.35

*AM* Amniotic membrane, *Av.* Average, *N* Normal delivery *CS* Caesarean delivery, *cfu* colony forming unit, *SD* standard deviation

* Placenta numbers are the actual numbers in the BTRC tissue bank records

In this study, four batches (A, B, C and D) were chosen. The AMs in each batch were prepared by ascertained couple of technicians (two technicians/one batch).

#### Bioburden estimation for validation and substantiation of 25 kGy RSD

The bioburden experiments were conducted according to ISO 11737-1 (2006). Bioburden estimation was done for each of the 10 selected AM grafts of the four AM batches. Extraction of microbes from AM graft was done by washing in 300 ml polysorbate saline solution (0.1 % tween 80) and mechanical shaking for 15 min, the solutions were filtered under vacuum through a 0.45 μm pore size, 47 mm diameter sterile cellulose nitrate membrane filters (Whatman^®^ Cat# 7187, Whatman Paper Limited, England), using (Millipore pump, Model: WP6122050, Millipore corp., USA), the filters were incubated onto Tryptic Soy Agar (TSA) medium “Casein soya bean digest agar” (Code# 610052 Liofilchems.r.l. Biotechnology products, Italy) at 34 °C using (FIRLABO.sa, type: P1407B, France) incubator, the colony forming units (cfu) were counted and selected colonies were sub-cultured, morphologically and microscopically identified using gram’s stain and stocked in agar tubes at room temperature for further species identification.

#### Obtaining VD_max_^25^

According to VD_max_^25^ Method of the ISO 11137-2 (2006), the dose verification was performed for each of the four batches A, B, C and D. The procedure requires another ten AM grafts, nearly the same size as those used in bioburden estimation. The whole sample was used in this destructive test, thus the sample item portion (SIP) = 1. Ten AM grafts resembling a single batch were irradiated at room temperature with verification doses obtained from Table 9 of ISO, 11137-1 (2006), for levels of average bioburden ≤1,000 cfu and verification sterility assurance level (SAL) = 10^−1^ (means, probability of 1 non sterile item out of 10). Irradiation of samples was done using gamma irradiator at the National Center for Sciences and Nuclear Techniques (CNSTN), Sedi Thabet, Tunis-Tunisia, and Amber Perspex dosimeters were used to measure absorbed doses by AM grafts. Each of the ten radiation treated AM sample (SIP = 1) of each of the four batches A-D was tested for sterility using the recommendations in ISO 11737-2 (2006). Briefly, autoclaved media bottles containing 300 ml Tryptic Soy Broth (TSB) “Casein soya bean digest broth” (Code# 610053 Liofilchems.r.l. Biotechnology products, Italy) were used to check the sterility of amnion grafts irradiated at the verification doses; AM samples were transferred under aseptic control to the TSB media bottles, the bottles containing amnion samples were incubated at 34 °C for 14–21 days.

## Results and discussion

### Bioburden estimation

Bioburden is a good measure for monitoring microbiological quality of the final products of the routine processing of AM (Yusof and Hassan 2007) and as a reliable indicator on the level of hygienic conditions at the AM processing site. Bioburden variations might occur due to differences in AM preparation and handling skills of the working staff. AM processing must be properly carried out by staff who have adequate training in clean or aseptic processing.

Validation and substantiation of RSD for AM is an essential step for establishment of an SOP. Based on the requirement set by the ISO 11137-1 (2006), it was possible to validate and substantiate the RSD using Method VD_max_^25^, as all the four batches tested had bioburden counts ≤1,000 cfu/graft. The average number of AM grafts gained from an individual processed membrane was 3.28 AM grafts/placenta with a range of 2.6–3.6 The overall average size of the AM grafts was 99.0 cm^2^. List of placenta source and bioburden counts is illustrated in Table 1. For bioburden estimation, each AM graft was used entirely (SIP = 1); thereof, application of the correction factor for bioburden counts is not a strict requirement in this work. The average bioburden for the batches A, B, C and D were 3.9, 13.9, 46.8 and 7.8 cfu/AM graft respectively.

Statistical analysis of bioburden results as shown in Table 1 using two way ANOVA indicated no significant differences (α = 1 %) in bioburden of the AM prepared by four different couples of the tissue bank technicians, meaning that all of the technicians have similar skills and were able to follow the written SOP for processing of air dried AM in consistent manner. Processing of air dried AM was initially learnt by trial and error; upon several trials the errors became minimum and the practice became perfect. The skills in preparation techniques especially AM dissection, cleaning and aseptic packaging as well as bioburden determination using membrane filtration method are like any other skill, can be improved through practice. The overall average bioburden of the four batches (40 AM grafts) tested in this work was 18.35 cfu (Table 1) which is much lower than that processed in the early trials of AM preparations at the BTRC experimental tissue bank in 2004 with average cfu of ~10^3^–10^4^) (Marsit et al. 2006).

To ensure safety and quality of the tissue grafts for use in clinical applications, continuous training is mandatory for personnel involved in any part of the tissue banking activity, especially when there is a need for change in procedures or new scientific knowledge (Kaminski et al. 2013). Most of the improvements were attributed to the review of the SOP e.g. changing the disinfecting treatment with 70 % alcohol to 0.05 % sodium hypochlorite solution, furthermore, workers recruited as permanent tissue bank staff have more skills than those temporarily assigned with different procedures, and their experience were impacted positively on the overall activity.

Additionally, considerable checkings on the processing methodology such as sterile gloving, gowning and personnel movements and work behavior are done in parallel with continuous monitoring and assessment, additionally, aseptic handling of AM, especially during cutting and packing inside the safety cabinet must be carried out in a slow and purposefully manner which has led to a distinct effect in lowering the bioburden counts of the recently produced air dried AM batches. Handling of AM grafts after drying should be strictly done using sterile tools.

However, knowing the potential sources of contamination is a step towards avoiding it. When personnel is expected to be a potential source of contamination during processing, then the mishandling intervention of them has to be minimized, hence, the risk of microbiological contamination of amnion graft will be minimized through aseptic technique.

In general, the quality of our AM grafts in terms of bioburden is relatively low when compared to similar AM grafts produced at recognized or regional experimental tissue banks; in Indonesia, for example, the yearly average bioburden for lyophilized AMs produced at Batan Research Tissue Bank (BRTB) was 120 cfu in 1997 and decreased to 57 cfu in 1998/product unit (Hilmy et al. 2000), whereas, in Malaysia, the bioburden counts on AM grafts were improved over years ranging from 85.2 in 1990 to 2.7 cfu/amnion graft in 1996 (Yusof and Hassan 2007; Yusof et al. 2007) whilst, a study at the tissue banking laboratory of Nuclear Research Centre of Algiers-Algeria, showed that the average bioburden from 10 lyophilized AM samples was 572 cfu (Djefal et al. 2007).

### Validation and substantiation of 25 kGy radiation sterilization dose

#### Obtaining VD_max_^25^and performing verification dose experiment

Following the ISO 11137-2 VD_max_^25^ method, the dose verification was performed for each of the four batches A, B, C and D, therefore, another ten AM, SIP = 1 (from the matching placenta used in bioburden estimation and were nearly the same sizes), were irradiated at room temperature with verification doses of 6.1, 7.0, 8.2 and 6.9 kGy respectively. Verification doses were again obtained from Table 9 of the foresaid ISO document (ISO 11137-2 2006) based on their bioburden counts determined earlier according to ISO 11737-1 (2006), i.e. the corresponding average bioburden for batches A, B, C and D were 3.9, 13.9, 46.8 and 7.8 cfu/AM graft respectively.

The results of the dose verification sterility test after 14 days incubation period at 34 °C, showed that the verification was accepted for all the batches A, B, C and D, as none of the test had two or more positive results (Table 2). This result demonstrated that quality system applied at the BTRC tissue bank is capable of producing AM grafts with acceptable levels of bioburden (e.g. 18.35 cfu), which is far less than the limit set by the ISO document no. 11137-2 (2006) which is <1,000 cfu. Therefore the 25 kGy RSD is substantiated as the reliable dose for sterilization of AM prepared at the BTRC tissue bank.

**Table 2:** Results of verification experiment

Batch	Average bioburden (cfu/graft)	*Verification dose (kGy)	Dose range obtained by dosimetry (kGy) (min–max)	Sterility test result	Verification result
A	3.9	6.1	5.70–7.32	1 +ve	Accepted
B	13.9	7.6	7.06–8.6	All −ve	Accepted
C	47.8	8.8	8.25–9.68	All −ve	Accepted
D	7.8	6.9	6.15–6.58	All −ve	Accepted

* Verification doses obtained from Table 9 of the ISO 11137-2(2006)

Positive = +ve, Negative = −ve

#### Effect of placental source

For bioburden estimation, the term “batch” used here resembles a number of placentae processed within a single month, including placentae from both normal and caesarean delivery. The type of delivery was not controlled in our experiment, however, to compare both placentae types from the bioburden point of view, the average bioburden was calculated for each group of AM used in this work and statistically analyzed.

From the 40 AM grafts used for bioburden estimation for the dose verification purpose of the four processing batches, 36 AM grafts obtained from 18 normal and 18 caesarean deliveries were selected for the comparison. To eliminate errors, three amniotic membranes with comparably higher bioburden counts that originated from one caesarean placenta (No.36) were excluded from these calculations, as it was revealed being coincidently contaminated by fungal airborne spores during packaging.

The average bioburden results of AM grafts prepared from normal and caesarean placentae were 9.6 cfu ± 7.4/AM graft (range 1–26 cfu) and 8.9 cfu ± 6.3/AM graft (range 0–21 cfu) respectively (Table 3). Statistical analysis using two way ANOVA shows no significant differences (α = 1 %) between the two sources of placenta (normal and caesarian section), and any differences were due to chance or coincidence, *P* value <0.01 was considered statistically significant.

**Table 3:** Average bioburden (cfu) on AM samples obtained from normal and caesarean deliveries

Type of delivery	Bioburden (cfu) on tested AM samples	Average bioburden ± SD
Normal (n = 18)		174/18 = 9.6 ± 7.4 cfu/sample
Caesarean (n = 18)		161/18 = 8.9 ± 6.3 cfu/sample

*cfu* colony forming unit, *SD* standard deviation

Despite that the bioburden from the four tested batches (40 AM grafts) which result in overall average bioburden of 18.35 cfu/AM graft, is low; the risk from bacterial contamination of vaginal delivery obtained placenta, is higher than that from caesarian delivery source; even though, contamination of placenta is unavoidable in either normal or caesarian delivery types, however, the bacterial species associated with vaginal delivery are pathogenic and arising from different sources, i.e., vagina, gut, skin and mouth flora (Adds et al. 2001). In addition, contamination by personnel mishandling during processing is less risky than vaginal delivery contamination, unless otherwise they have insufficient skills that helps in prevent cross contamination, or transmitting radiation resistant bacterial spores from working environment.

Air dried AM attached to sterile gauze is intended to be used for skin burns; therefore, massive quantities are required, hence, natural deliveries are abundantly available with less processing costs. Adds et. al. showed that the all 21 samples collected in his study, both caesarean and vaginal, were contaminated with 22 different bacterial species. On the other hand, a verified gamma radiation sterilization dose is sufficient to eliminate bacterial contamination in the range specified by the standards, <1,000 cfu.

## Conclusion

Regardless of type of delivery, if placenta is properly handled and AM is prepared according to SOP established for air dried AM, the bioburden counts will be very low, and hence, the 25 kGy radiation dose will be easily substantiated using VD_max_^25^ approach, providing that the product average bioburden should be below 1,000 cfu per product unit.

In addition, the low bioburden must be maintained and result reproducibility needs to be confirmed by repeating the dose validation work quarterly a year as recommended by the ISO standards. Quality and sterility of AM do not rely only on the 25 kGy terminal sterilization dose, an improved SOP for processing and personnel progressive training are equally important and would have better effects.

Although the bioburden test of AM final packed product is a good measure to evaluate its microbiological quality during routine processing in tissue banking; however, the bioburden test is incapable to confirm the source of contamination and could only assume it by correlating the isolated bacterial genera to its natural floral localization.

If AM production could be identified as a “Process”, then the process capability can be evaluated in a frequent manner using statistical approaches such as the process capability index (Cpk) using X bar/R-Charts and Pareto analysis to set limits and to look for process changes, thus, identifying the most possible causes of contamination “defects”. These require environmental monitoring of the processing site and tracking any changes in number and type of the contaminants by taking air and water samples, samples from washing solutions, swab samples from surfaces, packing materials, used tools, placenta and end product as well as monitoring skill of personnel involved in the AM production.
